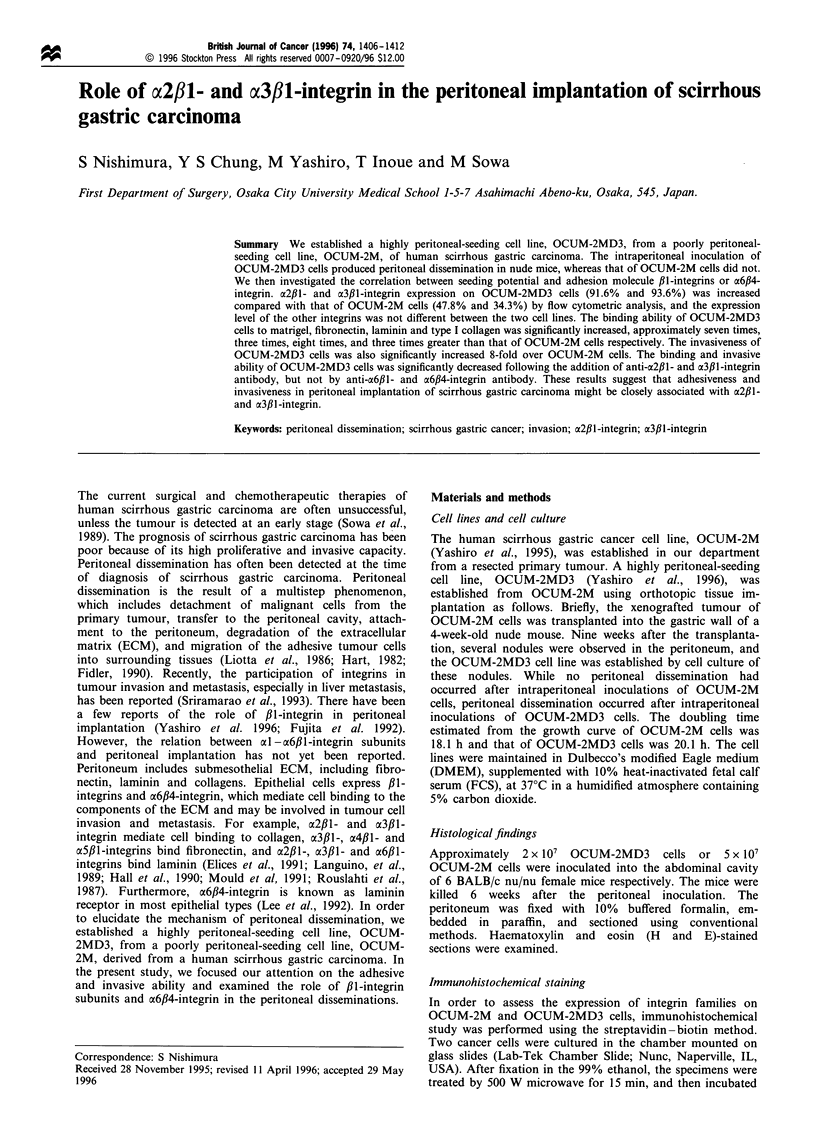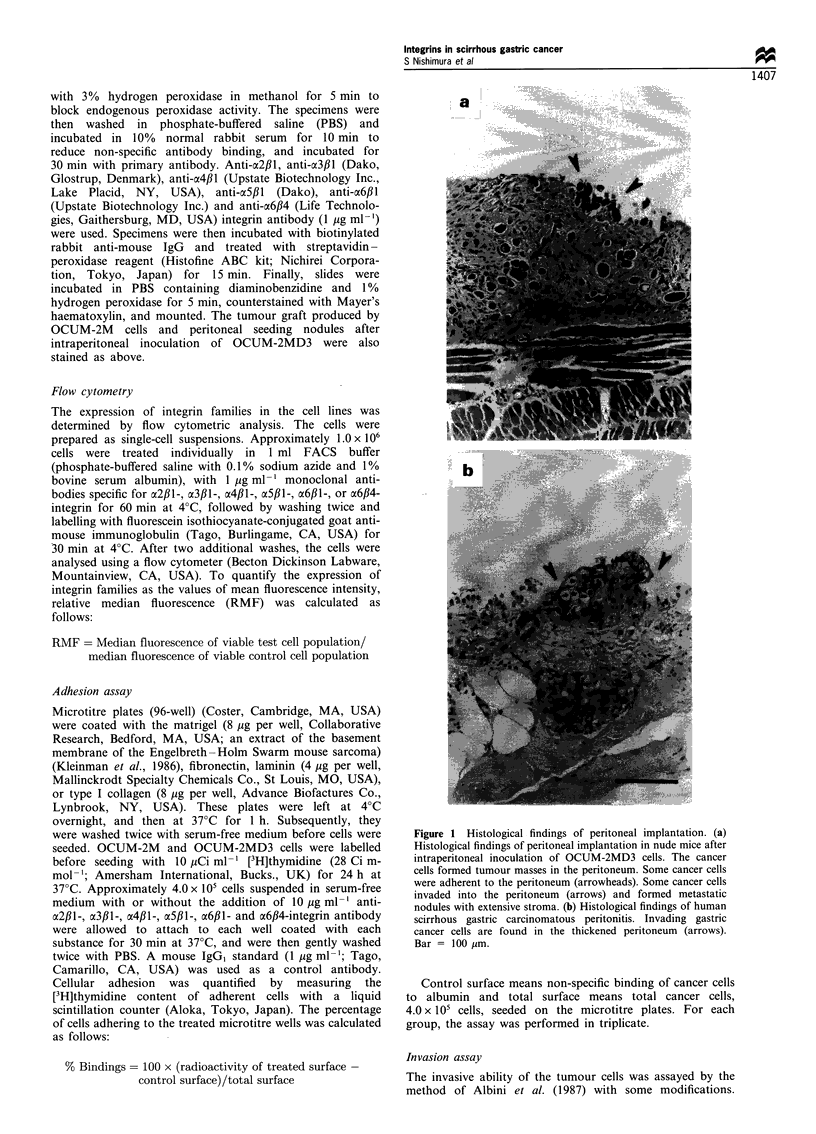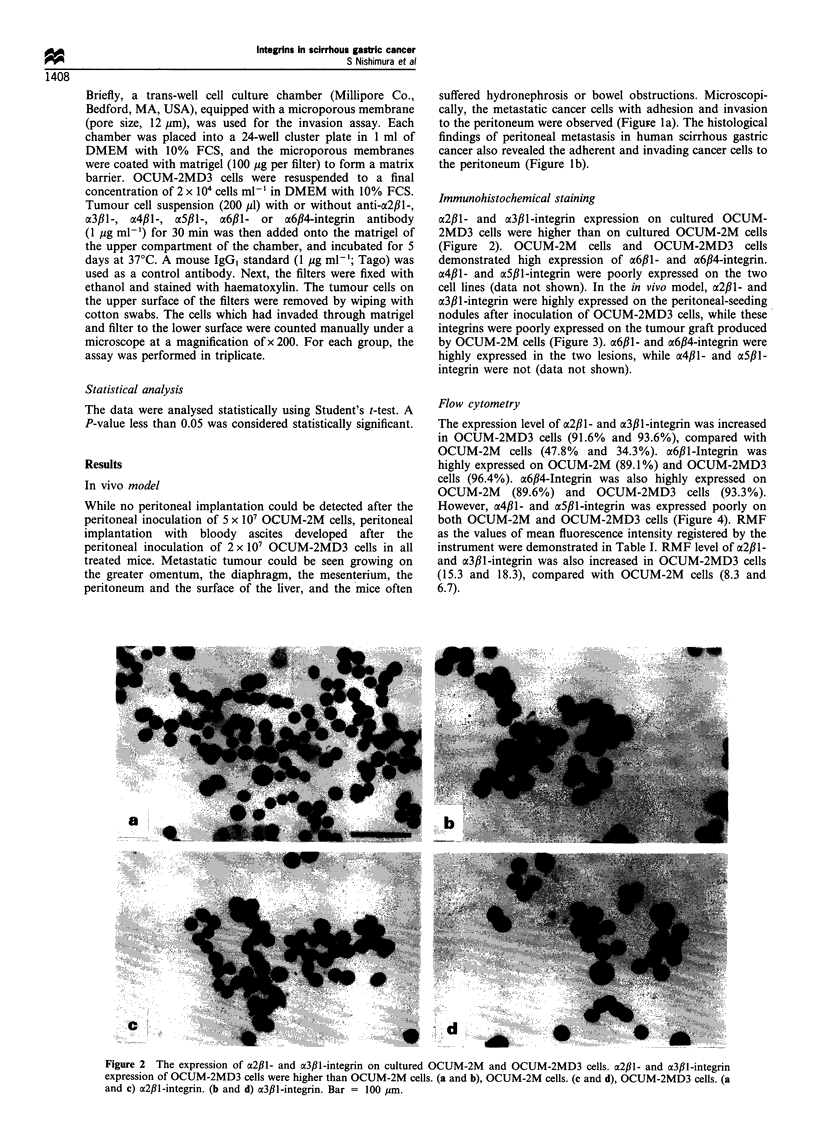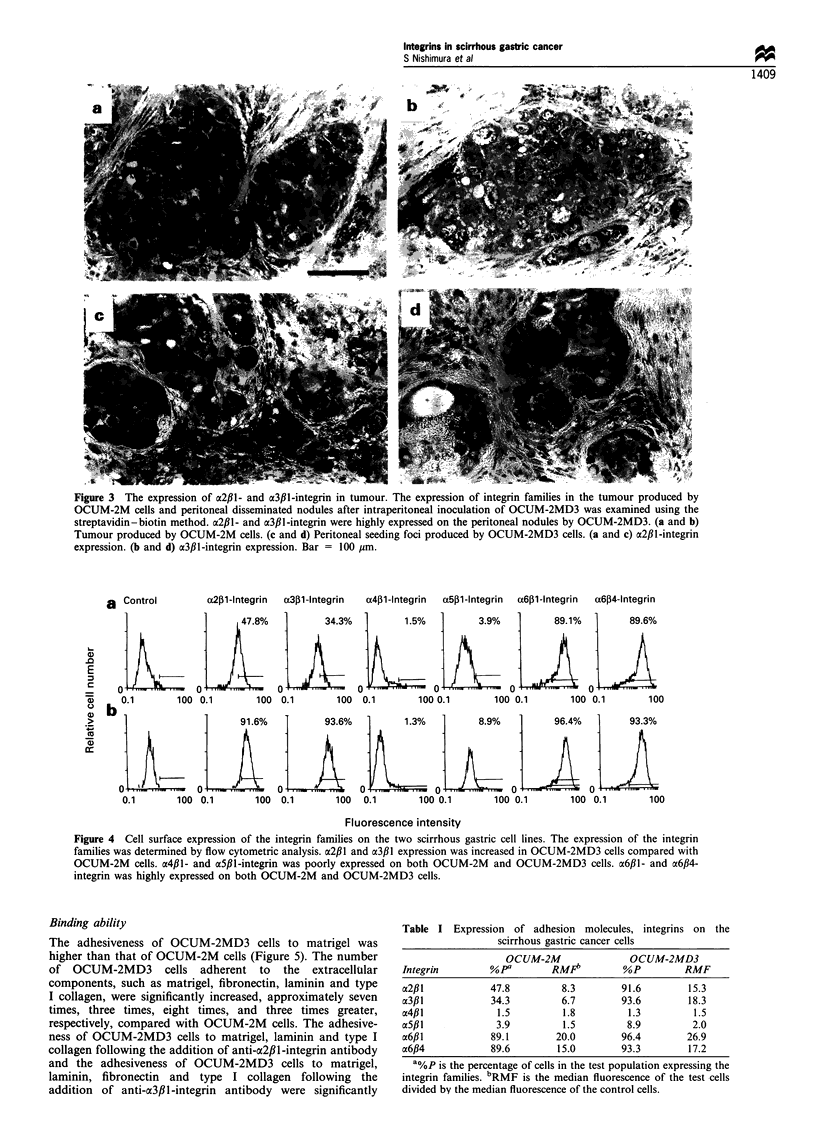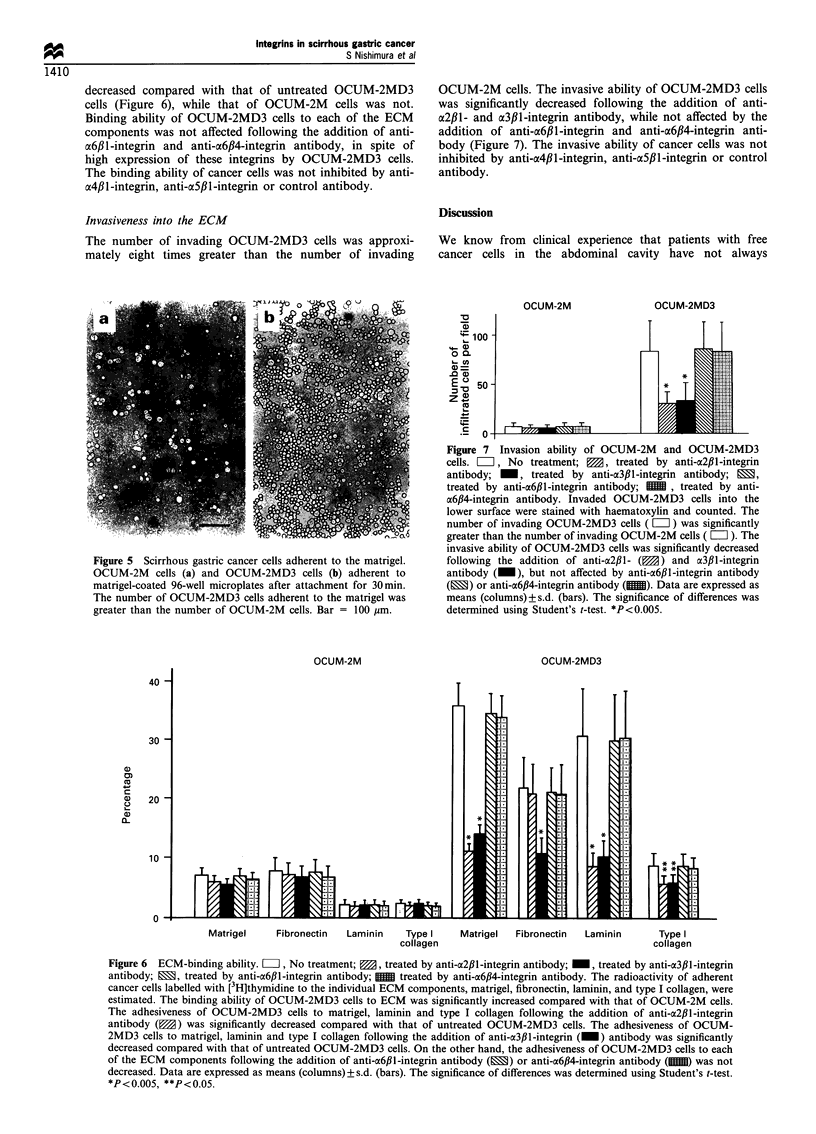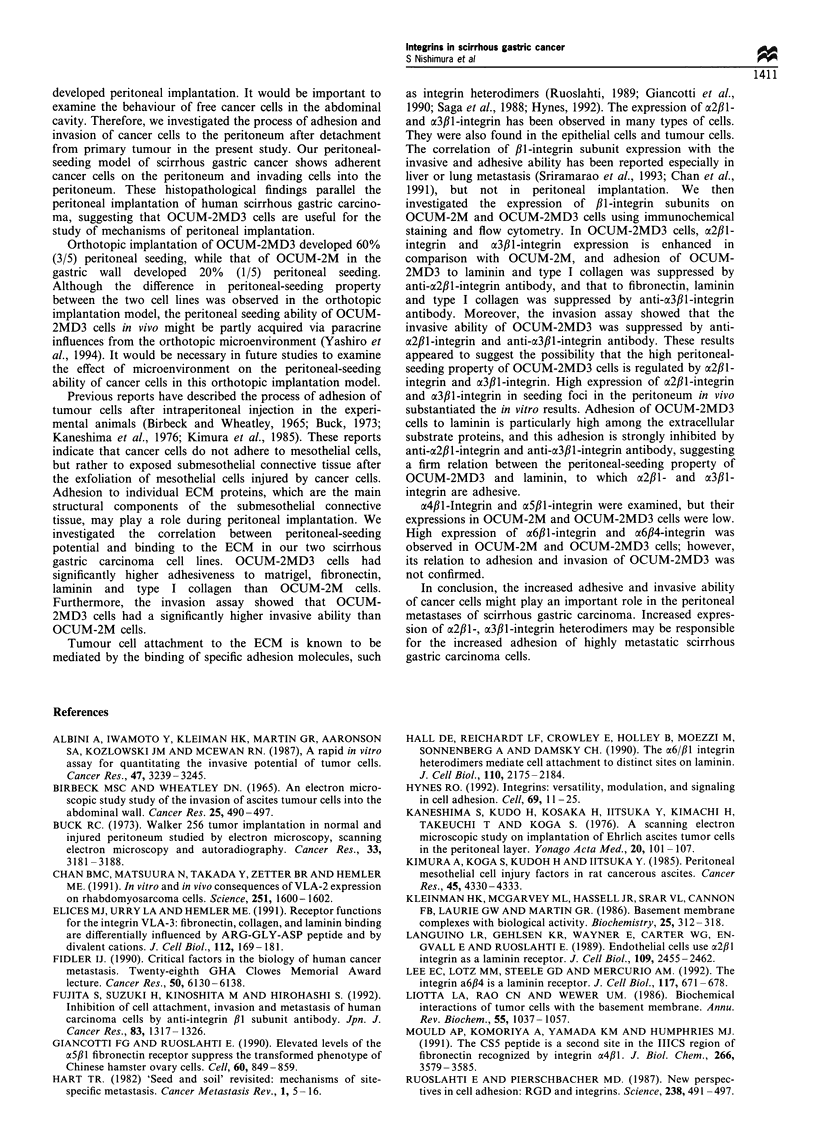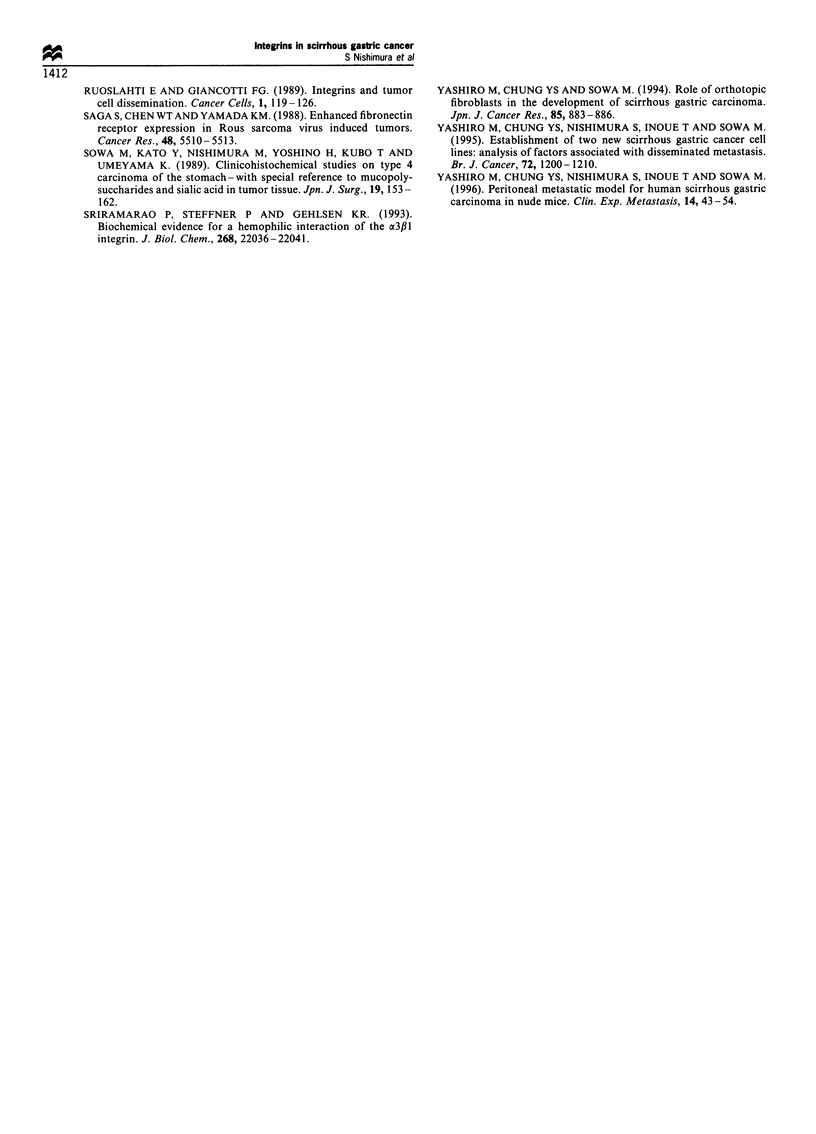# Role of alpha 2 beta 1- and alpha 3 beta 1-integrin in the peritoneal implantation of scirrhous gastric carcinoma.

**DOI:** 10.1038/bjc.1996.556

**Published:** 1996-11

**Authors:** S. Nishimura, Y. S. Chung, M. Yashiro, T. Inoue, M. Sowa

**Affiliations:** First Department of Surgery, Osaka City University Medical School, Japan.

## Abstract

**Images:**


British Journal of Cancer (1996) 74, 1406-1412
© 1996 Stockton Press All rights reserved 0007-0920/96 $12.00

### Role of α2β1- and α3β1-integrin in the peritoneal implantation of scirrhous gastric carcinoma

S Nishimura, Y S Chung, M Yashiro, T Inoue and M Sowa

*First Department of Surgery, Osaka City University Medical School 1-5-7 Asahimachi Abeno-ku, Osaka, 545, Japan.*

**Summary** We established a highly peritoneal-seeding cell line, OCUM-2MD3, from a poorly peritoneal-seeding cell line, OCUM-2M, of human scirrhous gastric carcinoma. The intraperitoneal inoculation of OCUM-2MD3 cells produced peritoneal dissemination in nude mice, whereas that of OCUM-2M cells did not. We then investigated the correlation between seeding potential and adhesion molecule β1-integrins or α6β4-integrin. α2β1- and α3β1-integrin expression on OCUM-2MD3 cells (91.6% and 93.6%) was increased compared with that of OCUM-2M cells (47.8% and 34.3%) by flow cytometric analysis, and the expression level of the other integrins was not different between the two cell lines. The binding ability of OCUM-2MD3 cells to matrigel, fibronectin, laminin and type I collagen was significantly increased, approximately seven times, three times, eight times, and three times greater than that of OCUM-2M cells respectively. The invasiveness of OCUM-2MD3 cells was also significantly increased 8-fold over OCUM-2M cells. The binding and invasive ability of OCUM-2MD3 cells was significantly decreased following the addition of anti-α2β1- and α3β1-integrin antibody, but not by anti-α6β1- and α6β4-integrin antibody. These results suggest that adhesiveness and invasiveness in peritoneal implantation of scirrhous gastric carcinoma might be closely associated with α2β1- and α3β1-integrin.

**Keywords:** peritoneal dissemination; scirrhous gastric cancer; invasion; α2β1-integrin; α3β1-integrin

The current surgical and chemotherapeutic therapies of human scirrhous gastric carcinoma are often unsuccessful, unless the tumour is detected at an early stage (Sowa *et al.*, 1989). The prognosis of scirrhous gastric carcinoma has been poor because of its high proliferative and invasive capacity. Peritoneal dissemination has often been detected at the time of diagnosis of scirrhous gastric carcinoma. Peritoneal dissemination is the result of a multistep phenomenon, which includes detachment of malignant cells from the primary tumour, transfer to the peritoneal cavity, attachment to the peritoneum, degradation of the extracellular matrix (ECM), and migration of the adhesive tumour cells into surrounding tissues (Liotta *et al.*, 1986; Hart, 1982; Fidler, 1990). Recently, the participation of integrins in tumour invasion and metastasis, especially in liver metastasis, has been reported (Sriramarao *et al.*, 1993). There have been a few reports of the role of β1-integrin in peritoneal implantation (Yashiro *et al.* 1996; Fujita *et al.* 1992). However, the relation between α1–α6β1-integrin subunits and peritoneal implantation has not yet been reported. Peritoneum includes submesothelial ECM, including fibronectin, laminin and collagens. Epithelial cells express β1-integrins and α6β4-integrin, which mediate cell binding to the components of the ECM and may be involved in tumour cell invasion and metastasis. For example, α2β1- and α3β1-integrin mediate cell binding to collagen, α3β1-, α4β1- and α5β1-integrins bind fibronectin, and α2β1-, α3β1- and α6β1-integrins bind laminin (Elices *et al.*, 1991; Languino, *et al.*, 1989; Hall *et al.*, 1990; Mould *et al*, 1991; Rouslahti *et al.*, 1987). Furthermore, α6β4-integrin is known as laminin receptor in most epithelial types (Lee *et al.*, 1992). In order to elucidate the mechanism of peritoneal dissemination, we established a highly peritoneal-seeding cell line, OCUM-2MD3, from a poorly peritoneal-seeding cell line, OCUM-2M, derived from a human scirrhous gastric carcinoma. In the present study, we focused our attention on the adhesive and invasive ability and examined the role of β1-integrin subunits and α6β4-integrin in the peritoneal disseminations.

Correspondence: S Nishimura
Received 28 November 1995; revised 11 April 1996; accepted 29 May 1996

### Materials and methods

### *Cell lines and cell culture*

The human scirrhous gastric cancer cell line, OCUM-2M (Yashiro *et al.*, 1995), was established in our department from a resected primary tumour. A highly peritoneal-seeding cell line, OCUM-2MD3 (Yashiro *et al.*, 1996), was established from OCUM-2M using orthotopic tissue implantation as follows. Briefly, the xenografted tumour of OCUM-2M cells was transplanted into the gastric wall of a 4-week-old nude mouse. Nine weeks after the transplantation, several nodules were observed in the peritoneum, and the OCUM-2MD3 cell line was established by cell culture of these nodules. While no peritoneal dissemination had occurred after intraperitoneal inoculations of OCUM-2M cells, peritoneal dissemination occurred after intraperitoneal inoculations of OCUM-2MD3 cells. The doubling time estimated from the growth curve of OCUM-2M cells was 18.1 h and that of OCUM-2MD3 cells was 20.1 h. The cell lines were maintained in Dulbecco's modified Eagle medium (DMEM), supplemented with 10% heat-inactivated fetal calf serum (FCS), at 37°C in a humidified atmosphere containing 5% carbon dioxide.

### *Histological findings*

Approximately $2\times10^7$ OCUM-2MD3 cells or $5\times10^7$ OCUM-2M cells were inoculated into the abdominal cavity of 6 BALB/c nu/nu female mice respectively. The mice were killed 6 weeks after the peritoneal inoculation. The peritoneum was fixed with 10% buffered formalin, embedded in paraffin, and sectioned using conventional methods. Haematoxylin and eosin (H and E)-stained sections were examined.

### *Immunohistochemical staining*

In order to assess the expression of integrin families on OCUM-2M and OCUM-2MD3 cells, immunohistochemical study was performed using the streptavidin–biotin method. Two cancer cells were cultured in the chamber mounted on glass slides (Lab-Tek Chamber Slide; Nunc, Naperville, IL, USA). After fixation in the 99% ethanol, the specimens were treated by 500 W microwave for 15 min, and then incubated with 3% hydrogen peroxidase in methanol for 5 min to block endogenous peroxidase activity. The specimens were then washed in phosphate-buffered saline (PBS) and incubated in 10% normal rabbit serum for 10 min to reduce non-specific antibody binding, and incubated for 30 min with primary antibody. Anti-$\alpha2\beta1$, anti-$\alpha3\beta1$ (Dako, Glostrup, Denmark), anti-$\alpha4\beta1$ (Upstate Biotechnology Inc., Lake Placid, NY, USA), anti-$\alpha5\beta1$ (Dako), anti-$\alpha6\beta1$ (Upstate Biotechnology Inc.) and anti-$\alpha6\beta4$ (Life Technologies, Gaithersburg, MD, USA) integrin antibody (1 $\mu$g ml$^{-1}$) were used. Specimens were then incubated with biotinylated rabbit anti-mouse IgG and treated with streptavidin–peroxidase reagent (Histofine ABC kit; Nichirei Corporation, Tokyo, Japan) for 15 min. Finally, slides were incubated in PBS containing diaminobenzidine and 1% hydrogen peroxidase for 5 min, counterstained with Mayer's haematoxylin, and mounted. The tumour graft produced by OCUM-2M cells and peritoneal seeding nodules after intraperitoneal inoculation of OCUM-2MD3 were also stained as above.

*Flow cytometry*

The expression of integrin families in the cell lines was determined by flow cytometric analysis. The cells were prepared as single-cell suspensions. Approximately $1.0 \times 10^6$ cells were treated individually in 1 ml FACS buffer (phosphate-buffered saline with 0.1% sodium azide and 1% bovine serum albumin), with 1 $\mu$g ml$^{-1}$ monoclonal antibodies specific for $\alpha2\beta1$-, $\alpha3\beta1$-, $\alpha4\beta1$-, $\alpha5\beta1$-, $\alpha6\beta1$-, or $\alpha6\beta4$-integrin for 60 min at 4°C, followed by washing twice and labelling with fluorescein isothiocyanate-conjugated goat anti-mouse immunoglobulin (Tago, Burlingame, CA, USA) for 30 min at 4°C. After two additional washes, the cells were analysed using a flow cytometer (Becton Dickinson Labware, Mountainview, CA, USA). To quantify the expression of integrin families as the values of mean fluorescence intensity, relative median fluorescence (RMF) was calculated as follows:

RMF = Median fluorescence of viable test cell population/ median fluorescence of viable control cell population

*Adhesion assay*

Microtitre plates (96-well) (Coster, Cambridge, MA, USA) were coated with the matrigel (8 $\mu$g per well, Collaborative Research, Bedford, MA, USA; an extract of the basement membrane of the Engelbreth–Holm Swarm mouse sarcoma) (Kleinman *et al.*, 1986), fibronectin, laminin (4 $\mu$g per well, Mallinckrodt Specialty Chemicals Co., St Louis, MO, USA), or type I collagen (8 $\mu$g per well, Advance Biofactures Co., Lynbrook, NY, USA). These plates were left at 4°C overnight, and then at 37°C for 1 h. Subsequently, they were washed twice with serum-free medium before cells were seeded. OCUM-2M and OCUM-2MD3 cells were labelled before seeding with 10 $\mu$Ci ml$^{-1}$ [$^3$H]thymidine (28 Ci mmol$^{-1}$; Amersham International, Bucks., UK) for 24 h at 37°C. Approximately $4.0 \times 10^5$ cells suspended in serum-free medium with or without the addition of 10 $\mu$g ml$^{-1}$ anti-$\alpha2\beta1$-, $\alpha3\beta1$-, $\alpha4\beta1$-, $\alpha5\beta1$-, $\alpha6\beta1$- and $\alpha6\beta4$-integrin antibody were allowed to attach to each well coated with each substance for 30 min at 37°C, and were then gently washed twice with PBS. A mouse $IgG_1$ standard (1 $\mu$g ml$^{-1}$; Tago, Camarillo, CA, USA) was used as a control antibody. Cellular adhesion was quantified by measuring the [$^3$H]thymidine content of adherent cells with a liquid scintillation counter (Aloka, Tokyo, Japan). The percentage of cells adhering to the treated microtitre wells was calculated as follows:

% Bindings = 100 × (radioactivity of treated surface − control surface)/total surface

**Figure 1** Histological findings of peritoneal implantation. (**a**) Histological findings of peritoneal implantation in nude mice after intraperitoneal inoculation of OCUM-2MD3 cells. The cancer cells formed tumour masses in the peritoneum. Some cancer cells were adherent to the peritoneum (arrowheads). Some cancer cells invaded into the peritoneum (arrows) and formed metastatic nodules with extensive stroma. (**b**) Histological findings of human scirrhous gastric carcinomatous peritonitis. Invading gastric cancer cells are found in the thickened peritoneum (arrows). Bar = 100 $\mu$m.

Control surface means non-specific binding of cancer cells to albumin and total surface means total cancer cells, $4.0 \times 10^5$ cells, seeded on the microtitre plates. For each group, the assay was performed in triplicate.

*Invasion assay*

The invasive ability of the tumour cells was assayed by the method of Albini *et al.* (1987) with some modifications.

Briefly, a trans-well cell culture chamber (Millipore Co., Bedford, MA, USA), equipped with a microporous membrane (pore size, 12 μm), was used for the invasion assay. Each chamber was placed into a 24-well cluster plate in 1 ml of DMEM with 10% FCS, and the microporous membranes were coated with matrigel (100 μg per filter) to form a matrix barrier. OCUM-2MD3 cells were resuspended to a final concentration of $2 \times 10^4$ cells $ml^{-1}$ in DMEM with 10% FCS. Tumour cell suspension (200 μl) with or without anti-α2β1-, α3β1-, α4β1-, α5β1-, α6β1- or α6β4-integrin antibody (1 μg $ml^{-1}$) for 30 min was then added onto the matrigel of the upper compartment of the chamber, and incubated for 5 days at 37°C. A mouse $IgG_1$ standard (1 μg $ml^{-1}$; Tago) was used as a control antibody. Next, the filters were fixed with ethanol and stained with haematoxylin. The tumour cells on the upper surface of the filters were removed by wiping with cotton swabs. The cells which had invaded through matrigel and filter to the lower surface were counted manually under a microscope at a magnification of × 200. For each group, the assay was performed in triplicate.

*Statistical analysis*

The data were analysed statistically using Student's *t*-test. A *P*-value less than 0.05 was considered statistically significant.

### Results

In vivo *model*

While no peritoneal implantation could be detected after the peritoneal inoculation of $5 \times 10^7$ OCUM-2M cells, peritoneal implantation with bloody ascites developed after the peritoneal inoculation of $2 \times 10^7$ OCUM-2MD3 cells in all treated mice. Metastatic tumour could be seen growing on the greater omentum, the diaphragm, the mesenterium, the peritoneum and the surface of the liver, and the mice often suffered hydronephrosis or bowel obstructions. Microscopically, the metastatic cancer cells with adhesion and invasion to the peritoneum were observed (Figure 1a). The histological findings of peritoneal metastasis in human scirrhous gastric cancer also revealed the adherent and invading cancer cells to the peritoneum (Figure 1b).

*Immunohistochemical staining*

α2β1- and α3β1-integrin expression on cultured OCUM-2MD3 cells were higher than on cultured OCUM-2M cells (Figure 2). OCUM-2M cells and OCUM-2MD3 cells demonstrated high expression of α6β1- and α6β4-integrin. α4β1- and α5β1-integrin were poorly expressed on the two cell lines (data not shown). In the *in vivo* model, α2β1- and α3β1-integrin were highly expressed on the peritoneal-seeding nodules after inoculation of OCUM-2MD3 cells, while these integrins were poorly expressed on the tumour graft produced by OCUM-2M cells (Figure 3). α6β1- and α6β4-integrin were highly expressed in the two lesions, while α4β1- and α5β1-integrin were not (data not shown).

*Flow cytometry*

The expression level of α2β1- and α3β1-integrin was increased in OCUM-2MD3 cells (91.6% and 93.6%), compared with OCUM-2M cells (47.8% and 34.3%). α6β1-Integrin was highly expressed on OCUM-2M (89.1%) and OCUM-2MD3 cells (96.4%). α6β4-Integrin was also highly expressed on OCUM-2M (89.6%) and OCUM-2MD3 cells (93.3%). However, α4β1- and α5β1-integrin was expressed poorly on both OCUM-2M and OCUM-2MD3 cells (Figure 4). RMF as the values of mean fluorescence intensity registered by the instrument were demonstrated in Table I. RMF level of α2β1- and α3β1-integrin was also increased in OCUM-2MD3 cells (15.3 and 18.3), compared with OCUM-2M cells (8.3 and 6.7).

**Figure 2** The expression of α2β1- and α3β1-integrin on cultured OCUM-2M and OCUM-2MD3 cells. α2β1- and α3β1-integrin expression of OCUM-2MD3 cells were higher than OCUM-2M cells. (**a** and **b**), OCUM-2M cells. (**c** and **d**), OCUM-2MD3 cells. (**a** and **c**) α2β1-integrin. (**b** and **d**) α3β1-integrin. Bar = 100 μm.

**Figure 3** The expression of α2β1- and α3β1-integrin in tumour. The expression of integrin families in the tumour produced by OCUM-2M cells and peritoneal disseminated nodules after intraperitoneal inoculation of OCUM-2MD3 was examined using the streptavidin–biotin method. α2β1- and α3β1-integrin were highly expressed on the peritoneal nodules by OCUM-2MD3. (**a** and **b**) Tumour produced by OCUM-2M cells. (**c** and **d**) Peritoneal seeding foci produced by OCUM-2MD3 cells. (**a** and **c**) α2β1-integrin expression. (**b** and **d**) α3β1-integrin expression. Bar = 100 μm.

**Figure 4** Cell surface expression of the integrin families on the two scirrhous gastric cell lines. The expression of the integrin families was determined by flow cytometric analysis. α2β1 and α3β1 expression was increased in OCUM-2MD3 cells compared with OCUM-2M cells. α4β1- and α5β1-integrin was poorly expressed on both OCUM-2M and OCUM-2MD3 cells. α6β1- and α6β4-integrin was highly expressed on both OCUM-2M and OCUM-2MD3 cells.

### *Binding ability*

The adhesiveness of OCUM-2MD3 cells to matrigel was higher than that of OCUM-2M cells (Figure 5). The number of OCUM-2MD3 cells adherent to the extracellular components, such as matrigel, fibronectin, laminin and type I collagen, were significantly increased, approximately seven times, three times, eight times, and three times greater, respectively, compared with OCUM-2M cells. The adhesiveness of OCUM-2MD3 cells to matrigel, laminin and type I collagen following the addition of anti-α2β1-integrin antibody and the adhesiveness of OCUM-2MD3 cells to matrigel, laminin, fibronectin and type I collagen following the addition of anti-α3β1-integrin antibody were significantly

**Table I** Expression of adhesion molecules, integrins on the scirrhous gastric cancer cells

| | *OCUM-2M* | | *OCUM-2MD3* | |
|---|---|---|---|---|
| *Integrin* | *%P*[a] | *RMF*[b] | *%P* | *RMF* |
| α2β1 | 47.8 | 8.3 | 91.6 | 15.3 |
| α3β1 | 34.3 | 6.7 | 93.6 | 18.3 |
| α4β1 | 1.5 | 1.8 | 1.3 | 1.5 |
| α5β1 | 3.9 | 1.5 | 8.9 | 2.0 |
| α6β1 | 89.1 | 20.0 | 96.4 | 26.9 |
| α6β4 | 89.6 | 15.0 | 93.3 | 17.2 |

[a]*%P* is the percentage of cells in the test population expressing the integrin families. [b]RMF is the median fluorescence of the test cells divided by the median fluorescence of the control cells.

decreased compared with that of untreated OCUM-2MD3 cells (Figure 6), while that of OCUM-2M cells was not. Binding ability of OCUM-2MD3 cells to each of the ECM components was not affected following the addition of anti-$\alpha6\beta1$-integrin and anti-$\alpha6\beta4$-integrin antibody, in spite of high expression of these integrins by OCUM-2MD3 cells. The binding ability of cancer cells was not inhibited by anti-$\alpha4\beta1$-integrin, anti-$\alpha5\beta1$-integrin or control antibody.

*Invasiveness into the ECM*

The number of invading OCUM-2MD3 cells was approximately eight times greater than the number of invading OCUM-2M cells. The invasive ability of OCUM-2MD3 cells was significantly decreased following the addition of anti-$\alpha2\beta1$- and $\alpha3\beta1$-integrin antibody, while not affected by the addition of anti-$\alpha6\beta1$-integrin and anti-$\alpha6\beta4$-integrin antibody (Figure 7). The invasive ability of cancer cells was not inhibited by anti-$\alpha4\beta1$-integrin, anti-$\alpha5\beta1$-integrin or control antibody.

### Discussion

We know from clinical experience that patients with free cancer cells in the abdominal cavity have not always

**Figure 5** Scirrhous gastric cancer cells adherent to the matrigel. OCUM-2M cells (**a**) and OCUM-2MD3 cells (**b**) adherent to matrigel-coated 96-well microplates after attachment for 30 min. The number of OCUM-2MD3 cells adherent to the matrigel was greater than the number of OCUM-2M cells. Bar = 100 μm.

**Figure 7** Invasion ability of OCUM-2M and OCUM-2MD3 cells. ▭, No treatment; ▨, treated by anti-$\alpha2\beta1$-integrin antibody; ■, treated by anti-$\alpha3\beta1$-integrin antibody; ▧, treated by anti-$\alpha6\beta1$-integrin antibody; ▦, treated by anti-$\alpha6\beta4$-integrin antibody. Invaded OCUM-2MD3 cells into the lower surface were stained with haematoxylin and counted. The number of invading OCUM-2MD3 cells (▭) was significantly greater than the number of invading OCUM-2M cells (▭). The invasive ability of OCUM-2MD3 cells was significantly decreased following the addition of anti-$\alpha2\beta1$- (▨) and $\alpha3\beta1$-integrin antibody (■), but not affected by anti-$\alpha6\beta1$-integrin antibody (▧) or anti-$\alpha6\beta4$-integrin antibody (▦). Data are expressed as means (columns) ± s.d. (bars). The significance of differences was determined using Student's *t*-test. $^{*}P<0.005$.

**Figure 6** ECM-binding ability. ▭, No treatment; ▨, treated by anti-$\alpha2\beta1$-integrin antibody; ■, treated by anti-$\alpha3\beta1$-integrin antibody; ▧, treated by anti-$\alpha6\beta1$-integrin antibody; ▦ treated by anti-$\alpha6\beta4$-integrin antibody. The radioactivity of adherent cancer cells labelled with [$^3$H]thymidine to the individual ECM components, matrigel, fibronectin, laminin, and type I collagen, were estimated. The binding ability of OCUM-2MD3 cells to ECM was significantly increased compared with that of OCUM-2M cells. The adhesiveness of OCUM-2MD3 cells to matrigel, laminin and type I collagen following the addition of anti-$\alpha2\beta1$-integrin antibody (▨) was significantly decreased compared with that of untreated OCUM-2MD3 cells. The adhesiveness of OCUM-2MD3 cells to matrigel, laminin and type I collagen following the addition of anti-$\alpha3\beta1$-integrin (■) antibody was significantly decreased compared with that of untreated OCUM-2MD3 cells. On the other hand, the adhesiveness of OCUM-2MD3 cells to each of the ECM components following the addition of anti-$\alpha6\beta1$-integrin antibody (▧) or anti-$\alpha6\beta4$-integrin antibody (▦) was not decreased. Data are expressed as means (columns) ± s.d. (bars). The significance of differences was determined using Student's *t*-test. $^{*}P<0.005$, $^{**}P<0.05$.

developed peritoneal implantation. It would be important to examine the behaviour of free cancer cells in the abdominal cavity. Therefore, we investigated the process of adhesion and invasion of cancer cells to the peritoneum after detachment from primary tumour in the present study. Our peritoneal-seeding model of scirrhous gastric cancer shows adherent cancer cells on the peritoneum and invading cells into the peritoneum. These histopathological findings parallel the peritoneal implantation of human scirrhous gastric carcinoma, suggesting that OCUM-2MD3 cells are useful for the study of mechanisms of peritoneal implantation.

Orthotopic implantation of OCUM-2MD3 developed 60% (3/5) peritoneal seeding, while that of OCUM-2M in the gastric wall developed 20% (1/5) peritoneal seeding. Although the difference in peritoneal-seeding property between the two cell lines was observed in the orthotopic implantation model, the peritoneal seeding ability of OCUM-2MD3 cells *in vivo* might be partly acquired via paracrine influences from the orthotopic microenvironment (Yashiro *et al.*, 1994). It would be necessary in future studies to examine the effect of microenvironment on the peritoneal-seeding ability of cancer cells in this orthotopic implantation model.

Previous reports have described the process of adhesion of tumour cells after intraperitoneal injection in the experimental animals (Birbeck and Wheatley, 1965; Buck, 1973; Kaneshima *et al.*, 1976; Kimura *et al.*, 1985). These reports indicate that cancer cells do not adhere to mesothelial cells, but rather to exposed submesothelial connective tissue after the exfoliation of mesothelial cells injured by cancer cells. Adhesion to individual ECM proteins, which are the main structural components of the submesothelial connective tissue, may play a role during peritoneal implantation. We investigated the correlation between peritoneal-seeding potential and binding to the ECM in our two scirrhous gastric carcinoma cell lines. OCUM-2MD3 cells had significantly higher adhesiveness to matrigel, fibronectin, laminin and type I collagen than OCUM-2M cells. Furthermore, the invasion assay showed that OCUM-2MD3 cells had a significantly higher invasive ability than OCUM-2M cells.

Tumour cell attachment to the ECM is known to be mediated by the binding of specific adhesion molecules, such as integrin heterodimers (Ruoslahti, 1989; Giancotti *et al.*, 1990; Saga *et al.*, 1988; Hynes, 1992). The expression of $\alpha2\beta1$- and $\alpha3\beta1$-integrin has been observed in many types of cells. They were also found in the epithelial cells and tumour cells. The correlation of $\beta1$-integrin subunit expression with the invasive and adhesive ability has been reported especially in liver or lung metastasis (Sriramarao *et al.*, 1993; Chan *et al.*, 1991), but not in peritoneal implantation. We then investigated the expression of $\beta1$-integrin subunits on OCUM-2M and OCUM-2MD3 cells using immunochemical staining and flow cytometry. In OCUM-2MD3 cells, $\alpha2\beta1$-integrin and $\alpha3\beta1$-integrin expression is enhanced in comparison with OCUM-2M, and adhesion of OCUM-2MD3 to laminin and type I collagen was suppressed by anti-$\alpha2\beta1$-integrin antibody, and that to fibronectin, laminin and type I collagen was suppressed by anti-$\alpha3\beta1$-integrin antibody. Moreover, the invasion assay showed that the invasive ability of OCUM-2MD3 was suppressed by anti-$\alpha2\beta1$-integrin and anti-$\alpha3\beta1$-integrin antibody. These results appeared to suggest the possibility that the high peritoneal-seeding property of OCUM-2MD3 cells is regulated by $\alpha2\beta1$-integrin and $\alpha3\beta1$-integrin. High expression of $\alpha2\beta1$-integrin and $\alpha3\beta1$-integrin in seeding foci in the peritoneum *in vivo* substantiated the *in vitro* results. Adhesion of OCUM-2MD3 cells to laminin is particularly high among the extracellular substrate proteins, and this adhesion is strongly inhibited by anti-$\alpha2\beta1$-integrin and anti-$\alpha3\beta1$-integrin antibody, suggesting a firm relation between the peritoneal-seeding property of OCUM-2MD3 and laminin, to which $\alpha2\beta1$- and $\alpha3\beta1$-integrin are adhesive.

$\alpha4\beta1$-Integrin and $\alpha5\beta1$-integrin were examined, but their expressions in OCUM-2M and OCUM-2MD3 cells were low. High expression of $\alpha6\beta1$-integrin and $\alpha6\beta4$-integrin was observed in OCUM-2M and OCUM-2MD3 cells; however, its relation to adhesion and invasion of OCUM-2MD3 was not confirmed.

In conclusion, the increased adhesive and invasive ability of cancer cells might play an important role in the peritoneal metastases of scirrhous gastric carcinoma. Increased expression of $\alpha2\beta1$-, $\alpha3\beta1$-integrin heterodimers may be responsible for the increased adhesion of highly metastatic scirrhous gastric carcinoma cells.

### References

ALBINI A, IWAMOTO Y, KLEIMAN HK, MARTIN GR, AARONSON SA, KOZLOWSKI JM AND MCEWAN RN. (1987), A rapid *in vitro* assay for quantitating the invasive potential of tumor cells. *Cancer Res.*, **47**, 3239–3245.

BIRBECK MSC AND WHEATLEY DN. (1965). An electron microscopic study study of the invasion of ascites tumour cells into the abdominal wall. *Cancer Res.* **25**, 490–497.

BUCK RC. (1973). Walker 256 tumor implantation in normal and injured peritoneum studied by electron microscopy, scanning electron microscopy and autoradiography. *Cancer Res.*, **33**, 3181–3188.

CHAN BMC, MATSUURA N, TAKADA Y, ZETTER BR AND HEMLER ME. (1991). *In vitro* and *in vivo* consequences of VLA-2 expression on rhabdomyosarcoma cells. *Science*, **251**, 1600–1602.

ELICES MJ, URRY LA AND HEMLER ME. (1991). Receptor functions for the integrin VLA-3: fibronectin, collagen, and laminin binding are differentially influenced by ARG-GLY-ASP peptide and by divalent cations. *J. Cell Biol.*, **112**, 169–181.

FIDLER IJ. (1990). Critical factors in the biology of human cancer metastasis. Twenty-eighth GHA Clowes Memorial Award lecture. *Cancer Res.*, **50**, 6130–6138.

FUJITA S, SUZUKI H, KINOSHITA M AND HIROHASHI S. (1992). Inhibition of cell attachment, invasion and metastasis of human carcinoma cells by anti-integrin $\beta1$ subunit antibody. *Jpn. J. Cancer Res.*, **83**, 1317–1326.

GIANCOTTI FG AND RUOSLAHTI E. (1990). Elevated levels of the $\alpha5\beta1$ fibronectin receptor suppress the transformed phenotype of Chinese hamster ovary cells. *Cell*, **60**, 849–859.

HART TR. (1982) 'Seed and soil' revisited: mechanisms of site-specific metastasis. *Cancer Metastasis Rev.*, **1**, 5–16.

HALL DE, REICHARDT LF, CROWLEY E, HOLLEY B, MOEZZI M, SONNENBERG A AND DAMSKY CH. (1990). The $\alpha6/\beta1$ integrin heterodimers mediate cell attachment to distinct sites on laminin. *J. Cell Biol.*, **110**, 2175–2184.

HYNES RO. (1992). Integrins: versatility, modulation, and signaling in cell adhesion. *Cell*, **69**, 11–25.

KANESHIMA S, KUDO H, KOSAKA H, IITSUKA Y, KIMACHI H, TAKEUCHI T AND KOGA S. (1976). A scanning electron microscopic study on implantation of Ehrlich ascites tumor cells in the peritoneal layer. *Yonago Acta Med.*, **20**, 101–107.

KIMURA A, KOGA S, KUDOH H AND IITSUKA Y. (1985). Peritoneal mesothelial cell injury factors in rat cancerous ascites. *Cancer Res.*, **45**, 4330–4333.

KLEINMAN HK, MCGARVEY ML, HASSELL JR, SRAR VL, CANNON FB, LAURIE GW AND MARTIN GR. (1986). Basement membrane complexes with biological activity. *Biochemistry*, **25**, 312–318.

LANGUINO LR, GEHLSEN KR, WAYNER E, CARTER WG, ENGVALL E AND RUOSLAHTI E. (1989). Endothelial cells use $\alpha2\beta1$ integrin as a laminin receptor. *J. Cell Biol.*, **109**, 2455–2462.

LEE EC, LOTZ MM, STEELE GD AND MERCURIO AM. (1992). The integrin a$6\beta4$ is a laminin receptor. *J. Cell Biol.*, **117**, 671–678.

LIOTTA LA, RAO CN AND WEWER UM. (1986). Biochemical interactions of tumor cells with the basement membrane. *Annu. Rev. Biochem.*, **55**, 1037–1057.

MOULD AP, KOMORIYA A, YAMADA KM AND HUMPHRIES MJ. (1991). The CS5 peptide is a second site in the IIICS region of fibronectin recognized by integrin $\alpha4\beta1$. *J. Biol. Chem.*, **266**, 3579–3585.

RUOSLAHTI E AND PIERSCHBACHER MD. (1987). New perspectives in cell adhesion: RGD and integrins. *Science*, **238**, 491–497.

RUOSLAHTI E AND GIANCOTTI FG. (1989). Integrins and tumor cell dissemination. *Cancer Cells*, **1**, 119–126.

SAGA S, CHEN WT AND YAMADA KM. (1988). Enhanced fibronectin receptor expression in Rous sarcoma virus induced tumors. *Cancer Res.*, **48**, 5510–5513.

SOWA M, KATO Y, NISHIMURA M, YOSHINO H, KUBO T AND UMEYAMA K. (1989). Clinicohistochemical studies on type 4 carcinoma of the stomach–with special reference to mucopolysuccharides and sialic acid in tumor tissue. *Jpn. J. Surg.*, **19**, 153–162.

SRIRAMARAO P, STEFFNER P AND GEHLSEN KR. (1993). Biochemical evidence for a hemophilic interaction of the $\alpha3\beta1$ integrin. *J. Biol. Chem.*, **268**, 22036–22041.

YASHIRO M, CHUNG YS AND SOWA M. (1994). Role of orthotopic fibroblasts in the development of scirrhous gastric carcinoma. *Jpn. J. Cancer Res.*, **85**, 883–886.

YASHIRO M, CHUNG YS, NISHIMURA S, INOUE T AND SOWA M. (1995). Establishment of two new scirrhous gastric cancer cell lines: analysis of factors associated with disseminated metastasis. *Br. J. Cancer*, **72**, 1200–1210.

YASHIRO M, CHUNG YS, NISHIMURA S, INOUE T AND SOWA M. (1996). Peritoneal metastatic model for human scirrhous gastric carcinoma in nude mice. *Clin. Exp. Metastasis*, **14**, 43–54.